# Development of a Mobile Phone Addiction Craving Scale and Its Validation in a Spanish Adult Population

**DOI:** 10.3389/fpsyt.2017.00090

**Published:** 2017-05-30

**Authors:** José De-Sola, Hernán Talledo, Gabriel Rubio, Fernando Rodríguez de Fonseca

**Affiliations:** ^1^Department of Psychobiology, School of Psychology, Complutense University of Madrid, Madrid, Spain; ^2^San Ignacio de Loyola University, Lima, Peru; ^3^University of the Pacific in Lima, Lima, Peru; ^4^Department of Psychiatry, Complutense University of Madrid, Research Institute i+12, Addictive Disorder Network (RETIS), 12 de Octubre University Hospital of Madrid, Madrid, Spain; ^5^Clinical Mental Health Management Unit, Instituto IBIMA, Hospital Regional Universitario de Malaga, Malaga, Spain

**Keywords:** mobile phone addiction, MPPUS, problem phone use, cell phone use, craving

## Abstract

In some people, problematic cell phone use can lead to situations in which they lose control, similar to those observed in other cases of addiction. Although different scales have been developed to assess its severity, we lack an instrument that is able to determine the desire or craving associated with it. Thus, with the objective of evaluating craving for cell phone use, in this study, we develop and present the Mobile Phone Addiction Craving Scale (MPACS). It consists of eight Likert-style items, with 10 response options, referring to possible situations in which the interviewee is asked to evaluate the degree of restlessness that he or she feels if the cell phone is unavailable at the moment. It can be self-administered or integrated in an interview when abuse or problems are suspected. With the existence of a single dimension, reflected in the exploratory factor analysis (EFA), the scale presents adequate reliability and internal consistency (α = 0.919). Simultaneously, we are able to show significantly increased correlations (*r* = 0.785, *p* = 0.000) with the Mobile Phone Problematic Use Scale (MPPUS) and state anxiety (*r* = 0.330, *p* = 0.000). We are also able to find associations with impulsivity, measured using the urgency, premeditation, perseverance, and sensation seeking scale, particularly in the dimensions of negative urgency (*r* = 0.303, *p* = 0.000) and positive urgency (*r* = 0.290, *p* = 0.000), which confirms its construct validity. The analysis of these results conveys important discriminant validity among the MPPUS user categories that are obtained using the criteria by Chow et al. (1). The MPACS demonstrates higher levels of craving in persons up to 35 years of age, reversing with age. In contrast, we do not find significant differences among the sexes. Finally, a receiver operating characteristic (ROC) analysis allows us to establish the scores from which we are able to determine the different levels of craving, from the absence of craving to that referred to as addiction. Based on these results, we can conclude that this scale is a reliable tool that complements ongoing studies on problematic cell phone use.

## Introduction

Cell phone abuse has given rise to important issues that have been extensively described in the literature, including physical conditions such as muscle pain and eye problems (2, 3) and psychological conditions such as auditory and tactile delusions (4, 5), in addition to behavioral consequences such as anxiety, insomnia, and disturbances in personal, family, and work life (6–9).

Although a large portion of studies has focused attention on the student and adolescent population, we know that mobile phone addiction also affects large portions of the adult population (10). Nonetheless, for a while, there has been a discussion on whether cell phone abuse can be considered an addiction, similar to the current perception of gambling. However, even beyond its approximation with it, the concept of behavioral addiction is increasingly expanding, by a large number of researchers and to a broad range of behaviors such as the Internet, shopping, and food. These addictions arise in turn as comparisons with symptoms of drug use such as alcohol, tobacco, and other substances.

There is no common consensus and unanimity regarding the concept of craving; however, it is turning out to be crucial in the explanation of an addiction to a point that in the new *Diagnostic and statistical manual for mental disorders (5th ed.)*, craving is incorporated to all drug use disorders as one of the essential diagnostic criteria (11). To put simply, craving can be defined as an unstoppable and uncontrollable desire that can lead to use (a drug, a technology), despite its negative and detrimental effects. This impulsive loss-of-control can lead to a search for gratification or positive reinforcement or turn into a compulsion whose objective would be to avoid distress or dysphoria, a negative reinforcement that would sustain the abnormal use (12).

Merikle (13) studies the subjective perception of affected individuals and indicates that craving is expressed through yearning or the intense desire for something, relating consumption and relapse with the intensity of the craving. Thus, craving would arise as much from internal stimuli, such as feelings and thoughts, as from external or environmental stimuli. It also differs depending on the type of substance or drug (12, 13), in which cravings for cocaine or alcohol would not be the same. In all likelihood, in behavioral addictions, a different craving must exist among the behaviors. Furthermore, two types of craving have been described: the anhedonic, which results out of boredom, and the conditioned, which is associated with circumstances, instances, or prior stimuli (14), in all cases being an essential subjective and motivational–emotional state that does not sufficiently explain the use (15).

At this time, the majority of studies and craving scale constructions have focused on drug or substance use, which, given the cognitive nature of this use, have been based on self-reports, as in the case of cannabis (16), alcohol (17), nicotine (18), cocaine (19–21), and, recently, inhalables (22). Nonetheless, we do not have many tools for the evaluation of craving in behavioral addictions, except food and chocolate addictions, in which there is a certain profusion of studies and scales that are very much related to eating problems (23–28).

In the case of the cell phone, when one talks about craving, the following symptoms are described: urgency, abstinence, dependency, difficulty of control, increased use (29–31), the need to stay connected, with irritability and restlessness if the phone is unavailable or if one is not allowed to use it (8, 32–35), and some stress and mood changes (36, 37). Recently, addiction and craving have also been described with specific applications such as Facebook (38–41). In some cases, these cravings coexist with substance abuse, such as alcohol (42) and with anxiety, impulsivity, or depression; even in psychiatric contexts, it has been observed that anxiety and depression can foster craving in general (43).

Thus, given the existence of craving in problematic mobile phone use and the lack of specific evaluation tools, the objective of this study is based on the development of the Mobile Phone Addiction Craving Scale (MPACS) after confirming its construct and discriminant internal validity.

Although in the present study we equate that which some investigators refer to as problematic cell phone use with addiction, we are aware of the limitations derived of a lack of agreement on whether addiction to technology (i.e., internet, cell phone) fulfills or not this equivalence to recognized addictions. We have launched this research to gain insights on the established criteria underlying recognized drug use disorders, addressing the monitoring of craving as a key aspect of a potential mobile phone addiction. The development of a craving scale, such as the present MPACS, will help to further investigate in alternative populations the existence of this important criterion incorporated in major drug use disorders and certain behavioral addictions. If finally problematic mobile phone use is agreed to be considered an addiction, identification and scoring of craving will be an important instrument to monitor how this novel addiction affects the population on risk, specially adolescents, in which the impulsive loss-of-control and the fascination with new technologies is greater.

## Materials and Methods

### Procedure

This study was performed using a questionnaire. The questionnaire was distributed between January and December 2014, *via* an online platform, which sent out invitations by email with directions arising from a database. Each participant received a link that allowed access to the interview *via* Sawtooth Software’s SSI Web Edition 6.8 program. With this program, the interviewee can take a break and return to the questionnaire at his or her leisure. The link was disabled once the interview was completed. All participants had to own their own cell phone as a condition for the study; this ownership was established by a filter question at the beginning that ended the interview if the answer was no.

Approximately 20% of the sample was obtained by our own mailings. The rest was completed using a database belonging to hired company specializing in sociological studies and online research that was composed of 151,170 people in Spain. The sample size was closed at 1,126 questionnaires out of 1,600 final mailings.

The total questionnaire takes approximately 20 min to complete, including several sections of a broader investigation. Only the results referring to the MPACS are presented in this study. We used adaptations of the Mobile Phone Problematic Use Scale (MPPUS) by Bianchi and Phillips (44) and the psychological factors of state anxiety and impulsivity as criteria for result validity, based on which we obtained the construct and discriminant internal validity of the MPACS.

### Tools Used

In addition to the MPACS, we included the tools with which external validation criteria have been linked.

#### Mobile Phone Addiction Craving Scale

The MPACS can be self-administered and consists of eight Likert-type items that range from 1 to 10 points with regard to the conformity of the statements. The possible range of scores would thus be 8–80, with an average of 22.85 and an SD of 14.85 (Table 1). The scale explores the main familiar situations where urges to use the mobile phone or anxiety derived of its unavailability may emerge.

**Table 1:** **Descriptive analysis and internal consistency of the items using Cronbach’s alpha coefficient and the factor loads from exploratory factor analysis (EFA)**.

	Range	Minimum	Maximum	Mean	SD	Median	Cronbach’s alpha if one component was eliminated	Factorial loads in EFA
P32—if I wanted to turn it on right now and could not or would not be allowed to	9	1	10	2.94	2.345	2.00	0.904	0.835
P32—if, at this very moment, I found myself out of battery or without coverage	9	1	10	2.92	2.351	2.00	0.906	0.818
P32—if, at this very moment, I should be forced to turn it off because I was at the movies or at work	9	1	10	1.85	1.717	1.00	0.916	0.716
P32—if, at this very moment, I realized that I left it at home	9	1	10	3.88	2.758	3.00	0.914	0.757
P32—if, at this very moment, I could not or if they did not let me reply to a message	9	1	10	2.78	2.285	2.00	0.906	0.824
P32—if I was with people at the moment I was using it and it did not work for me	9	1	10	2.70	2.298	1.00	0.907	0.814
P32—if I was in a place or a situation in which I always used it and no longer could	9	1	10	3.18	2.400	2.00	0.904	0.839
P32—if, at this very moment, I was restless and needed to relax and did not have it available	9	1	10	2.60	2.327	1.00	0.908	0.806
Total	72	8	80	22.85	14.85	18.00		

*Average values, SDs, and ranges are presented, both for the items and for the total amount. Cronbach’s alpha is presented for items assuming that one component was eliminated*.

The MPACS is presented as a fast evaluation tool for craving with regard to cell phone addiction, evaluating the degree of apprehension in eight possible situations in which one would not be able to use it. All situations refer to a hypothetical present moment (See Table 1 and the spanish version of MPACS scale in Appendix 1, supplementary material). We partially followed the format and style of the Spanish scale of cocaine craving developed by Durán and Becoña (20) in its construction due to its concision and use of ratings of the current moment. However, we did not include references to preceding days or anticipatory thoughts or cognitive constructs of use, given that, in an unprecedented manner, cell phones are integral parts of our everyday life, which does not allow choice of use as an option. We constructed the scale trying to reduce the number of items to facilitate its use. Language used is simple, avoiding technical words. The design selected was not a test–retest model so we did not generate a larger pool of items to reach the final structure of the scale.

#### Mobile Phone Problematic Use Scale (MPPUS)

We evaluated problematic cell phone use with the MPPUS (44), following our adaptation of the MPPUS to the Spanish adult population (in press), which in turn was based on research by López-Fernández et al. (45) among the adolescent population. In this adaptation, we categorized users following the criteria by Chow et al. (1), which involve the establishment of four categories of the MPPUS (casual users, habitual or regular users, at-risk users, and problematic users), using the percentiles of 15, 80, and 95.

This adaptation of the MPPUS consists of 26 Likert-type items, with a response range from 1 (not at all true) to 10 (completely true). In a prior exploratory factor analysis (EFA), we obtained four factors, of which, for usefulness, we only consider the first three, given that the last factor only contained one item. A total of 55.8% of the variance was explained by those three factors and relate to abuse and dependence (Factor I), craving and loss-of-control (Factor II), and dependency on the social environment (Factor III).

#### STAI-S and UPPS-P

As criteria of external validity, we also related the results of the MPACS with the state anxiety of the State-Trait Anxiety Inventory (STAI-S) (46) in its Spanish adaptation (47) and with impulsivity, measured by the urgency, premeditation, perseverance, and sensation seeking (UPPS) (48) in its development of five dimensions (UPPS-P) (49–51) and Spanish adaptation by Verdejo-García et al. (52).

State anxiety versus trait anxiety refers to transitional moments or periods characterized by tension, apprehension, and increased activity of the autonomic nervous system, which can vary in time or intensity. We have used state anxiety versus trait anxiety because it refers to the present time, in principle more objectifiable, assuming that state anxiety may also be reflected in trait anxiety. The STAI-S has 20 items with Likert-type scales ranging from 0 to 3 and a possible range of 0–60 points; some items are written in reverse, which was corrected in the final statistical analyses.

The UPPS consists of 59 items with Likert-type scales ranging from 1 to 4, depending on the level of agreement. It has five dimensions: negative urgency, which expresses the tendency to experience strong impulses under conditions of negative or dysphoric affective states; positive urgency, or the tendency to act hastily in response to positive emotional states; lack of premeditation, characterized by the lack of reflection or anticipation prior to the consequences of the behaviors themselves; lack of perseverance, or the difficulty in focusing on a task even though it is long, difficult, or boring; and sensation seeking, which may include both the tendency to seek and enjoy exciting activities and openness to new experiences, although in some cases they can be dangerous, thus having positive and negative aspects.

### Ethics Statement

The present project was approved by the Ethics Committee of i+12 Institute in Madrid. The study is a web platform-based survey, which includes an inform consent at the beginning of the questionnaire where the participants are informed of the purpose of the study. All subjects gave their free acceptance in accordance with the Declaration of Helsinki. All the procedures guarantee the generation of completely anonymized datasets.

### Statistical Analysis

Statistical analyses were performed using the SPSS program, v.23 and v.24 (IBM, Armonk, NY, USA). These analyses considered an evaluation of internal validity using EFA, addressing factor analysis by using orthogonal varimax rotation. In addition, we performed an evaluation of the construct by means of its relationship with external criteria, a discriminant evaluation using one way analysis of variance (ANOVA) (53), in the determination of differences and discrimination among the groups considered in the analysis and its reliability and consistency using the Cronbach’s alpha statistic (54). We planned a structural equation modeling for analyzing the results of the questionnaire. However, in the models of structural equations, we start with a theoretical model where the relations between the constructs are expressed by a set of regression equations. The factor analysis performed indicates the existence of a single construct that anticipated a failure of this model. Confirming this, we applied the structural equation modeling and found that the validation parameters of the model CFI = 0.939; RMSEA = 0.124; SRMR = 0.040 did not reach the validation limits (CFI > 0.95, RMSEA < 0.08 and SRMR < 0.10) (see Supplementary Material for additional information).

As criteria of external validity criteria, we calculated the Pearson correlations (55), measurements of the linear relationship between two quantitative variables of the MPACS with the psychological variables of state anxiety and impulsivity and with the problematic use or addiction measured by the MPPUS.

Finally, we used the technique of the receiver operating characteristic (ROC) curve, also using SPSS v.23. The ROC curve, designed as a system of decision-making, works with the concepts of sensitivity and specificity in a context of binary classifications of the detection of characteristics with regard to different levels or cutoff points (56). In our case, we used it in the determination of the cutoff points for the MPACS in the four user categories of the MPPUS.

Throughout the study, the accepted maximum level of significance was 5%.

### Sample and Participants

The sample consisted of 1,126 interviewees from throughout country, men and women between 16 and 65 years of age. We performed the sample selection using a non-probabilistic quota system with geographic proportionality to the population size in each of the 17 autonomous communities in Spain (except Ceuta and Melilla), based on 2014 data form the National Institute for Statistics. Slightly more than half of the interviews were performed in capitals of provinces and cities with more than 100,000 inhabitants (54.8%), with the rest being supplied by rural areas and smaller urban centers (45.2%).

Geographical areas as well as gender and age were established using a quota system. Age was mostly represented by blocks of 16- to 25-year olds and 26- to 35-year olds to obtain comparable information to other studies. The average age was 32.8 years, with an SD of 11.67. A total of 47.7% were male and 53.3% female, with mainly a higher level of education (63.5%).

## Results

### Reliability and Internal Consistency

Reliability and internal consistency were obtained using the Cronbach’s alpha total, by items and by halves (57).

In general terms, the MPACS presents with good internal consistency (α = 0.919). Similarly, the analysis by items indicates that in no cases, there is a value of less than 0.904, with a range of correlations of 0.904–0.916 (Table 1). The analysis by halves is provided; here, the correlations are calculated in two blocks, in our case, of four items, with coefficients of 0.841 and 0.884, respectively, in each half and with a Guttman coefficient of 0.887 or of covariation between both halves (58), which indicates that we are faced with items of great homogeneity.

### Internal Validity: EFA

In obtaining internal validity, we performed an EFA (59), following the method of principal components (60), with varimax rotation and Kaiser normalization (61).

The factor analysis confirms the unidimensionality of the scale, with one factor that explains 64.3% of the variance. All items maintain factor loads greater than 0.7, finding a range of 0.716–0.839 (Table 2), with the Kaiser–Meyer–Olkin (KMO) test (62) being the measure of sample adaptation (KMO = 0.920) and Bartlett’s test of sphericity (63) yielding a χ^2^ of 5,673.243 (df = 28; *p* < 0.000).

**Table 2:** **Correlations of the Mobile Phone Addiction Craving Scale (MPACS) with state anxiety, impulsivity, and the MPPUS**.

State anxiety and impulsivity with the MPACS	
State-Trait Anxiety Inventory (STAI-S)	0.330**
Total impulsivity (UPPS-S)	0.302**
Negative urgency	0.303**
Positive urgency	0.290**
Lack of premeditation	0.121**
Lack of perseverance	0.185**
Search for sensations	0.097**

**MPPUS with the MPACS**	

MPPUS–total	0.785**
Factor I—abuse and dependence	0.412**
Factor II—craving and loss-of-control	0.440**
Factor III—dependency on the social environment	0.542**

**Significant Pearson correlations at a level of 0.01.

### Construct Validity

In the determination of construct validity, state anxiety and impulsivity with its five dimensions and the problematic cell phone use of the MPPUS, both by total score and by its three factors, were used as external psychological criteria.

Thus, the total score of the MPACS maintains significant Pearson correlations with state anxiety and with the impulsivity dimensions of negative urgency and positive urgency (Table 2).

Similarly, its correlation with the sum of the MPPUS is elevated and significant, which confirms that the MPACS is closely related to the type of cell phone use. By factors, although significantly higher correlations with the three factors of the MPPUS are shown, it is greater with dependency on the social environment. Consequently, it is confirmed that an important aspect of craving cell phones would be related to social urgency and dependence (Table 2).

### Discriminant Validity: Differences by Users, Age, and Gender

When considering the average scores of the MPACS with regard to the abovementioned four categories of users utilized in the MPPUS (casual, regular, at-risk, and problematic), we can observe significant differences among these categories (Table 3). Thus, both the ANOVA (*F* = 336,261, *p* = 0.000) and the Kruskal–Wallis non-parametric test, which only considers inter-categorical differences (χ^2^ = 509,032, df = 3, *p* = 0.000), confirm that all of them differ significantly, which favors the discriminant validity of the MPACS in regard to the cell phone user type.

**Table 3:** **Mobile Phone Addiction Craving Scale (MPACS) scores for type of user of the MPPUS**.

	Casual	Regular	At-risk	Problematic	Total
Mean	9.33	19.98	37.82	50.57	22.85
SD	3.28	11.42	10.83	14.67	14.85
Median	8	17	39	49	18
Minimum score	8	8	8	10	8
Maximum score	33	65	72	80	80
Sample	153	742	173	58	1,126

*MPACS averages, SDs, medians, and minimum and maximum scores by type of users of MPPUS are shown*.

Concerning age, an ANOVA (64) shows that all groups significantly differ (*F* = 17,520, *p* = 0.000), with the scores, in relation to the mean, being the highest up to 35 years of age and reversing after that age (Table 4).

**Table 4:** **Mobile Phone Addiction Craving Scale (MPACS) scores for age categories**.

	16–25 years	26–35 years	36–45 years	46–55 years	56–65 years	Total
Mean	26.25	23.21	20.72	15.65	19.21	22.85
SD	15.13	15.63	13.97	13.97	13.40	14.85
Median	23	18	15	11	13	18
Minimum score	8	8	8	8	8	8
Maximum score	80	80	72	48	54	80
Sample	461	271	191	147	56	1,126

*MPACS averages, SDs, medians, and maximum and minimum scores by age groups are shown*.

Regarding gender, although men display a higher mean (M = 23.74, SD = 14.78) compared to women (M = 22.06, SD = 14.88) and compared to the total (M = 22.85, SD = 14.85), the differences are not significant, even though they are very close to the accepted maximum level of 5% in this study (*F* = 3,595, *p* = 0.058).

### ROC Analysis of the MPACS

Finally, the analysis of the ROC curve (56) allows us to determine the specificity and sensitivity of each of the MPPUS user categories and the ideal cutoff scores that determine the differences in craving between those groups. To that end, we determined the cutoff scores for each MPPUS user category by using the maximum possible criteria of sensitivity and specificity.

Thus, with 79.8% sensitivity and 80.7% specificity, we can consider that, starting at scores 30–31 of the MPACS, which correspond to the cutoff of at-risk users, one can speak about craving of risky cell phone use, whereas a score above 35–36 indicates levels of addiction with a sensitivity of 91.4% and a specificity of 82.1% (Table 5).

**Table 5:** **Receiver operating characteristic (ROC) curve and cutoff scores of the Mobile Phone Addiction Craving Scale for each category of user**.

	Cutoff score	Significance	Sensitivity (%)	Specificity (%)	Area and upper and lower limits
Casual users	8.50	0.000	32.7	10.7	0.110 (0.087–0.132)
Regular users	13.5	0.000	62.3	38.8	0.412 (0.371–0.454)
At-risk users	30.5	0.000	79.8	80.7	0.846 (0.819–0.872)
Problematic users	35.5	0.000	91.4	82.1	0.922 (0.884–0.960)

*The sensitivity, specificity, cutoff scores, area, and upper and lower limits on the ROC curve are shown*.

These data are consistent with the mean scores obtained among the groups of at-risk users (M = 37.82, SD = 10.83 of the MPACS) and problematic users (M = 50.7, SD = 14.67 of the MPACS).

## Discussion

In this study, we present the development of a mobile phone craving scale, the MPACS. This scale was developed in the context of a larger study, part of the growing evidence that cell phone abuse is or can give rise to a behavioral addiction that would affect broad sections of the population, just as is demonstrated in other studies (65–68).

The MPACS presents with adequate reliability and internal consistency, showing factorial unidimensionality; that is, it measures craving as a unique constructor, a result similar to that obtained for other drug scales (20, 22, 69). Although craving is considered a multifactorial construct for some researchers, the few items on the MPACS, such as its focus on a current situation, could have favored these results. Operationally, they indicate that we are measuring the possible degree of situational anxiety and restlessness when faced with absence of the cell phone or the inability to use the cell phone at the present time. We did not cover anticipatory thoughts or previous time stages, as other scales observe, and we are aware of this limitation. The lack of studies on problematic mobile phone use from the addiction perspective forced us to advance with caution because of the significant differences in between drug use disorders and behavioral disorders associated with problematic technology use.

Regarding construct validity, we used state anxiety and impulsivity as psychological references, on the one hand, and the problematic cell phone use of the MPPUS as criteria for cell phone addiction, on the other hand, although we are aware that there is not a clear agreement on the existence of mobile phone addiction in the scientific community.

The MPACS is significantly correlated with state anxiety and impulsivity, essentially in the dimensions of negative urgency and positive urgency in the latter. In general, landmark research shows that craving is related to anxiety or to impulsivity, though it is with the latter with which it has traditionally kept a close coexistence, particularly in its dimensions of urgency (70, 71) and difficulty in response inhibition (72), finding itself at the base of substance addictions, behavioral addictions in general and cell phone addiction in particular (73–75). Furthermore, anxiety also coexists with general addictions and with cell phone addiction in particular, an aspect that is widely recognized in various studies (32, 44, 76, 77).

Considering the framework of addiction, the MPACS demonstrates an increased Pearson correlation with the MPPUS, which confirms its construct validity with this concept; specifically, its greatest correlation is found with the factor of dependency on the social environment of the MPPUS. This finding indicates that cell phone addiction craving has a close relationship with anxiety and social dependency. This result is very much in line with those obtained in other studies (78–82).

Similarly, it reflects adequate discriminant validity by showing significant differences among the four user categories of the MPPUS (casual, regular, at-risk, and problematic). Consequently, it is confirmed that the scale has the capability of discriminating among cell phone users. Similarly, age reflects differences, with increased craving found in ages up to 35 years compared to the average and progressively reversing after that age. We cannot say the same for gender. Although men show higher scores of craving compared to women, the differences are not significant within the criteria established in this study, which sets a level of significance at 5%.

Finally, the sensitivity and specificity analyses using the ROC curve indicate that, starting at scores of 30–31 of the MPACS, which correspond to the cutoff scores for at-risk users, we can consider a user to exhibit craving with difficulties. Furthermore, a score above 35–36 indicates levels of craving befitting a true addiction and that correspond to the category of problematic users. As noted previously in another study (in press), we stated that the prevalence in the Spanish population is 20.5%, of whom 15.4% have problems or are at-risk users and 5.1% have problems that can be considered as having a cell phone addiction. The cutoff points of craving on the MPACS can be found in those groups.

To summarize, we consider not only that these results confirm the consideration that problematic mobile phone use is associated with craving in a similar way of major abused drugs. The MPACS scale proves to be a helpful and fast evaluation tool of cell phone craving in the general population. This tool can be employed in clinical settings to design cessation treatments in which subjects with very high scores should be using strategies based on coping and/or avoidance of cell phone use during the initial phase. Pharmacological management could simultaneously be required.

Naturally, more studies on behavioral addiction that outline the concept of craving in this context are needed, in addition to more studies on the similarities and differences of craving with the world of drugs. Just as in these studies, it is likely that different types of craving exist, just as there are various behaviors that are considered addictions. In addition to gender mainstreaming, other limitations of our study include the lack of a greater number of external criteria that would consolidate the construct validity and the fact that the MPACS, as a tool, would not always be able to be adjusted to the reality of the hypothetical present situations being evaluated.

## Ethics Statement

The study and protocols for recruitment were approved by the Ethics Committee of the Regional University Hospital of Málaga and were therefore conducted in accordance with the Declaration of Helsinki (seventh revision in 2013, Fortaleza, Brazil).

## Author Contributions

FF and GR designed the study that was developed by JG who did the survey and generated the database. HT did the statistical analysis. JG wrote the first draft of the manuscript that was corrected by all authors. FF obtained the financial support for this study.

## Conflict of Interest Statement

The authors declare that the research was conducted in the absence of any commercial or financial relationships that could be construed as a potential conflict of interest.
