# Supplementary material for: Development of a Mobile Phone Addiction Craving Scale and Its Validation in a Spanish Adult Population
Source: Front Psychiatry. 2017 May 30;8:90. doi: 10.3389/fpsyt.2017.00090 (PMC5447711; doi:10.3389/fpsyt.2017.00090)
Supplement: Supplementary file 1 [file Table_1.docx]

ANEXO I – ESCALA DE CRAVING DE ADICCION AL MOVIL (ECAM)

Por favor, ahora indica de la misma forma en una escala de 0 a 10, en donde **0 significa NADA y 10 MUCHO**, el **GRADO DE INQUIETUD Y ANSIEDAD** que te produciría **EN ESTE INSTANTE** cada uno de estos posibles momentos o situaciones en donde,si te encontrases **AHORA MISMO**, **no podrías utilizar tu teléfono móvil.**

1. **Ahora mismo, si quisiera encenderlo y no pudiera o no me lo permitiesen.**

1 2 3 4 5 6 7 8 9 10

Nada Mucho

1. **Si en este mismo momento me quedase sin batería o cobertura.**

1 2 3 4 5 6 7 8 9 10

Nada Mucho

1. **Si ahora me viese obligado a apagarlo porque estuviese en un cine o en el trabajo.**

1 2 3 4 5 6 7 8 9 10

Nada Mucho

1. **Si ahora mismo me diese cuenta de que se me ha olvidado en casa.**

1 2 3 4 5 6 7 8 9 10

Nada Mucho

1. **Ahora, si no pudiese o no me dejasen responder a un mensaje.**

1 2 3 4 5 6 7 8 9 10

Nada Mucho

1. **Si estuviese con gente en estos momentos que lo estuviese utilizando y a mi no me funcionase.**

1 2 3 4 5 6 7 8 9 10

Nada Mucho

1. **Si estuviera en un sitio o situación en donde siempre lo utilizo y ahora no pudiera.**

1 2 3 4 5 6 7 8 9 10

Nada Mucho

1. **Si en este momento estuviese inquieto y necesitase relajarme, y no lo tuviese a mano.**

1 2 3 4 5 6 7 8 9 10

Nada Mucho
