# Supplementary material for: Development of a Mobile Phone Addiction Craving Scale and Its Validation in a Spanish Adult Population
Source: Front Psychiatry. 2017 May 30;8:90. doi: 10.3389/fpsyt.2017.00090 (PMC5447711; doi:10.3389/fpsyt.2017.00090)
Supplement: Supplementary file 2 [file Data_Sheet_1.PDF]

Modelo de ecuaciones estructurales.

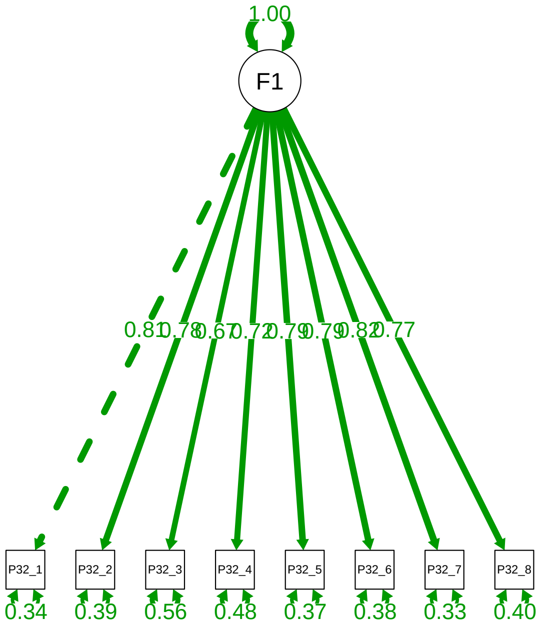


```
lavaan (0.5-23.1097) converged normally after  28 iterations

  Number of observations                          1126

  Estimator                                         ML
  Minimum Function Test Statistic              364.993
  Degrees of freedom                                20
  P-value (Chi-square)                           0.000

Model test baseline model:

  Minimum Function Test Statistic             5696.007
  Degrees of freedom                                28
  P-value                                        0.000

User model versus baseline model:

  Comparative Fit Index (CFI)                    0.939
```

```
  Tucker-Lewis Index (TLI)                       0.915

Loglikelihood and Information Criteria:

  Loglikelihood user model (H0)             -17589.721
  Loglikelihood unrestricted model (H1)     -17407.225

  Number of free parameters                         16
  Akaike (AIC)                               35211.443
  Bayesian (BIC)                             35291.866
  Sample-size adjusted Bayesian (BIC)        35241.045

Root Mean Square Error of Approximation:

  RMSEA                                          0.124
  90 Percent Confidence Interval          0.113  0.135
  P-value RMSEA <= 0.05                          0.000

Standardized Root Mean Square Residual:

  SRMR                                           0.040

Parameter Estimates:

  Information                                 Expected
  Standard Errors                             Standard

Latent Variables:
                   Estimate  Std.Err  z-value  P(>|z|)   Std.lv  Std.all
  F1 =~
    P32_r1            1.000                               1.899    0.810
    P32_r2            0.969    0.033   29.601    0.000    1.841    0.783
    P32_r3            0.602    0.025   23.979    0.000    1.143    0.666
    P32_r4            1.045    0.039   26.461    0.000    1.984    0.720
    P32_r5            0.955    0.032   30.154    0.000    1.814    0.794
    P32_r6            0.951    0.032   29.748    0.000    1.806    0.786
    P32_r7            1.035    0.033   31.479    0.000    1.965    0.819
    P32_r8            0.947    0.033   29.113    0.000    1.800    0.774

Variances:
                   Estimate  Std.Err  z-value  P(>|z|)   Std.lv  Std.all
   .P32_r1            1.887    0.094   19.971    0.000    1.887    0.343
   .P32_r2            2.134    0.104   20.614    0.000    2.134    0.387
   .P32_r3            1.639    0.074   22.173    0.000    1.639    0.557
   .P32_r4            3.663    0.169   21.626    0.000    3.663    0.482
   .P32_r5            1.929    0.095   20.381    0.000    1.929    0.370
   .P32_r6            2.016    0.098   20.554    0.000    2.016    0.382
   .P32_r7            1.895    0.096   19.722    0.000    1.895    0.329
   .P32_r8            2.171    0.104   20.804    0.000    2.171    0.401
    F1                3.607    0.223   16.149    0.000    1.000    1.000
```


FIMABIS
Avda. Jorge Luis Borges 15
Bloque 3 Planta 3 29010 Málaga
Tlf: +34 951440260
Fax: +34 951440263
web: http://www.imabis.org
e-mail: rita.perez@fimabis.org
